# Preliminary External Validation Results of the Artificial Intelligence-Based Headache Diagnostic Model: A Multicenter Prospective Observational Study

**DOI:** 10.3390/life14060744

**Published:** 2024-06-11

**Authors:** Mariko Okada, Masahito Katsuki, Tomokazu Shimazu, Takao Takeshima, Takashi Mitsufuji, Yasuo Ito, Katsumi Ohbayashi, Noboru Imai, Junichi Miyahara, Yasuhiko Matsumori, Yoshihiko Nakazato, Kazuki Fujita, Eri Hoshino, Toshimasa Yamamoto

**Affiliations:** 1 Department of Neurology, Saitama Medical University, 38 Morohongo, Moroyama-machi, Iruma-gun, Saitama 350-0495, Japan; mrk1030@saitama-med.ac.jp (M.O.);; 2 Physical Education and Health Center, Nagaoka University of Technology, Niigata 940-2137, Japan; 3 Department of Neurology, Saitama Neuropsychiatric Institute, Saitama 338-8577, Japan; 4 Headache Center and Department of Neurology, Tominaga Hospital, Osaka 556-0017, Japan; 5 Ohbayashi Clinic, Tochigi 321-0933, Japan; 6 Department of Neurology, Japanese Red Cross Shizuoka Hospital, Shizuoka 420-0853, Japan; 7 Sendai Headache and Neurology Clinic, Sendai 982-0014, Japan; 8 Department of Neurology, Jichi Medical University Saitama Medical Center, Saitama 330-8503, Japan

**Keywords:** artificial intelligence (AI), headache, machine learning, migraine, efficient medicine, digital transformation (DX), specialist, rural area, medication-overuse headache

## Abstract

The misdiagnosis of headache disorders is a serious issue, and AI-based headache model diagnoses with external validation are scarce. We previously developed an artificial intelligence (AI)-based headache diagnosis model using a database of 4000 patients’ questionnaires in a headache-specializing clinic and herein performed external validation prospectively. The validation cohort of 59 headache patients was prospectively collected from August 2023 to February 2024 at our or collaborating multicenter institutions. The ground truth was specialists’ diagnoses based on the initial questionnaire and at least a one-month headache diary after the initial consultation. The diagnostic performance of the AI model was evaluated. The mean age was 42.55 ± 12.74 years, and 51/59 (86.67%) of the patients were female. No missing values were reported. Of the 59 patients, 56 (89.83%) had migraines or medication-overuse headaches, and 3 (5.08%) had tension-type headaches. No one had trigeminal autonomic cephalalgias or other headaches. The models’ overall accuracy and kappa for the ground truth were 94.92% and 0.65 (95%CI 0.21–1.00), respectively. The sensitivity, specificity, precision, and F values for migraines were 98.21%, 66.67%, 98.21%, and 98.21%, respectively. There was disagreement between the AI diagnosis and the ground truth by headache specialists in two patients. This is the first external validation of the AI headache diagnosis model. Further data collection and external validation are required to strengthen and improve its performance in real-world settings.

## 1. Introduction

Primary headaches represent a prevalent issue in public health [1,2,3,4,5,6,7,8,9,10,11,12,13,14,15,16]. The International Classification of Headache Disorders, 3rd edition (ICHD-3) [17], indexes migraine [18,19,20], tension-type headache (TTH) [21,22,23], and trigeminal autonomic cephalalgias (TACs) [24] as typical examples of primary headaches, and they significantly impede patients’ everyday tasks. A correct diagnosis based on ICHD-3 criteria is essential for prescribing both preventative and acute treatment for headaches [25,26,27,28,29,30,31]. However, in 2023, only 982 headache specialists were practicing in Japan for more than 10 million people [18,19,20,32,33] experiencing migraines. Because of the low number of headache specialists, delays in headache diagnosis are likely, which can raise the risk of chronic migraines, treatment resistance, comorbidities, and medication-overuse headaches (MOHs) [32,34,35,36,37,38,39,40,41,42,43,44,45]. **Furthermore, late diagnosis and treatment initiation for migraines sometimes result in migraine prognoses ranging from episodic to chronic,** which are relatively difficult to treat [46,47,48,49]. In Japan, primary care physicians often handle the initial consultation for headache disorders, despite lacking specific training in this area. This situation places significant stress on primary care physicians and results in unmet medical needs. Similar challenges may also arise in other countries [50,51,52,53].

Artificial intelligence (AI)-based medical devices are gradually coming into the world. This is expected to reduce medical treatment costs and time [54,55,56,57]. AI-based solutions for headache diagnosis can address this issue because they can save time that would otherwise be taken up by medical interviews while increasing diagnostic performance [58,59,60]. If this AI becomes widely used, it could increase rational and efficient actions, such as patients who have not seen a doctor going to a hospital when they realize they have migraines, or non-specialists accurately diagnosing migraines in a short time and referring them to a specialist. However, only seven models [60,61,62,63,64,65,66] can diagnose primary headache subtypes and have been evaluated in both training and test datasets during development [67]. Furthermore, no models have performed external validation so far, and there are no reports examining whether AI performs the same capabilities in different domains as its training data.

Domain shifting is often a problem in AI development. AI that performs well on training data and target data used in the development environment will perform poorly in a different operating environment. This can occur when the probability distribution of target data in the operational environment differs from that in the development environment.

We previously developed an AI headache diagnosis model based on a headache-specialized outpatient dataset [65]. Because this AI was created using only data from headache outpatient clinics at one hospital, it is unknown whether it would perform similarly outside of that hospital. This means that the possibility of an inherent domain shift cannot be ruled out. In this study, we prospectively recruit headache patients and evaluate the AI’s performance compared to the headache specialists’ diagnoses as external validation. The objective is to prove that the diagnostic performance of AI is equivalent to that of a headache specialist.

## 2. Materials and Methods

### 2.1. AI Diagnosis Model

We utilized the AI headache diagnosis model that was previously reported and developed [65]. We will now provide a brief description of the model. It was constructed using data from 4000 consecutive patients aged 15 and over who visited the Tominaga Hospital Headache Center between March 2013 and December 2021. The patients’ questionnaire responses and diagnoses from their initial appointments were analyzed. Three headache specialists diagnosed all the patients. The 4000 patients were divided into training and test datasets randomly. The model was trained using 70% (2800) of the patients’ questionnaire data, which contained no missing information. To assess the model’s performance, it was tested on a separate dataset comprising responses from 1200 patients (30% of the total).

The headache questionnaire sheet had 17 variables. From the questionnaire sheet of each patient, we acquired 17 variables: age, sex, height, weight, age at headache onset, headache frequency, headache duration, site of headache, headache characteristics, headache severity, presence of aggravation by exercise, concomitant symptoms (including nausea, vomiting, photophobia, phonophobia, osmophobia, autonomic symptoms, paralysis), presence of aura, times when the headache is most likely to occur, inducement of headache, use of acute medication, and family history.

Headache diagnoses were classified into five categories as follows:

- Class 1: Migraines and MOHs (ICHD-3 codes 1 and 8.2);
- Class 2: TTHs (code 2);
- Class 3: TACs (code 3);
- Class 4: Other primary headaches (code 4);
- Class 5: Other headaches (other codes; secondary).

The average accuracy, sensitivity (recall), specificity, precision, and F values (Definition A: macro-average of F value for each class; Definition B: harmonic mean of average recall and average precision) were 76.25%, 56.26%, 92.16%, 61.24%, 56.88%, and 73.58%, respectively. The micro-average area under the curve of the receiver operating characteristic curve (AUC of the ROC) was 0.95.

### 2.2. Validation Cohort and Procedure

To test the model’s efficacy as external validation, we prospectively collected 59 headache patients from August 2023 to February 2024. Patient recruitment was carried out in 8 clinics and hospitals, and all of the patients consulted headache specialists in each facility. We validated the AI’s performance using 59 of the new patients’ data, including questionnaires and diagnoses by the specialists.

The recruitment procedure was as follows: Patients aged 20 or above with recurrent primary headaches who had undergone consultations at our institution or collaborating institutions for at least three months and consented to participate were included. A one-month observation period followed for electronic headache diary assessment before formal registration, and headache specialists confirmed their diagnoses based on both the initial headache questionnaire sheets and headache diary. The exclusion criteria were (i) pregnant or lactating individuals, those potentially pregnant during the study, or those wishing to become pregnant during the trial; (ii) patients with hemiplegic migraines; (iii) patients with severe cardiac, hepatic, renal, hematologic, or pulmonary disorders, or any condition deemed life-threatening by the investigator, **especially if the patients were taking a medication that has a side effect of headache or has a preventive effect of headaches, which may modify the nature of the headache characteristics**; (iv) patients with psychiatric disorders, including depression, following the Diagnostic and Statistical Manual of Mental Disorders (DSM)-5 diagnostic criteria; and (v) patients consuming more than 400 mg of caffeine per day, such as drinking 4 to 5 cups of coffee or regularly consuming caffeine-containing energy drinks.

The ground truth referred to the diagnoses provided by the headache specialists, and the agreement rates between headache specialists and AI diagnoses were assessed. Performance metrics such as accuracy, sensitivity (recall), specificity, precision, *F*-values (using definitions A and B), and kappa were evaluated to gauge performance.

### 2.3. Statistical Analysis

Variables with a normal distribution are presented as means (standard deviation), whereas those with a non-normal distribution are presented as medians (interquartile range). Unweighted Cohen’s kappa statistics were employed to gauge the agreement in diagnoses between the model and the raters. Unweighted kappa was chosen due to the nominal nature of the outcomes. The interpretation of kappa values followed Cohen’s guidelines, classifying agreement as “no agreement” for kappa ≤ 0, “none-to-slight agreement” for kappa = 0.01–0.20, “fair agreement” for kappa = 0.21–0.40, “moderate agreement” for kappa = 0.41–0.60, “substantial agreement” for kappa = 0.61–0.80, and “almost perfect agreement” for kappa = 0.81–1.00. The performance metrics of both the AI model and the raters were evaluated against specialist diagnoses, considered the reference standard (ground truth). Statistical analyses were conducted using SPSS 28.0.0 (IBM Corp., Armonk, NY, USA), Python 3.9.0, PyCaret 3.0.0, and Matplotlib 3.5.1.2.4.

### 2.4. Ethics

The Central Ethics Committee of Saitama Medical University approved this study (approval number: 2021-001). The requirement for written informed consent was obtained from all the patients. Opt-out consent documents were presented at the Tominaga Hospital and its website (https://www.tominaga.or.jp/about/registration_and_trial/ (accessed on 15 April 2024)) for patients who did not wish to participate. All methods in this study were performed following the relevant guidelines and regulations of the Declaration of Helsinki. This study was performed under the Strengthening the Reporting of Observational Studies in Epidemiology guidelines [68] and the Transparent Reporting of a Multivariable Prediction Model for Individual Prognosis or Diagnosis guidelines [69]. We also complied with the guidelines regarding medical AI development [67].

## 3. Results

### 3.1. Validation Cohort’s Characteristics

The diagnoses of the training, test [65], and external validation data are summarized in Table 1. The mean patient age was 42.55 (standard deviation 12.74) years, and 51/59 (86.67%) of the patients were female. No missing values were reported. Of the 59 patients, 56 (94.92%) had migraines or MOHs and 3 (5.08%) had TTHs. No one had TACs or other headaches (secondary headaches).

### 3.2. External Validation Performance

As the specialists’ diagnoses were ground truths, the AI’s performance was evaluated using the same patients’ questionnaire sheets. The overall accuracy and kappa for the ground truth were 94.92% and 0.65 (95%CI 0.21–1.00), respectively. The other indices, such as sensitivity, specificity, precision, and F values for migraine, were 98.21%, 66.67%, 98.21%, and 98.21%, respectively (Table 2).

There was disagreement between the AI diagnosis and the ground truth by the headache specialists in two patients. Among those, the AI misdiagnosed one TTH case as a migraine case and one migraine case as TACs.

**The former case was a 23-year-old female** with intermittent headaches for the past 6 years. The headache persisted throughout the day and every day. The center of her head ached. Sometimes, the headache pulsated, and sometimes it was persistent and tight. The pain intensity was moderate, and the headache was not aggravated by physical activity. It was accompanied by nausea, vomiting, and light–sound sensitivity. There was no aura. Stress increased the headaches’ intensity, and analgesics were used about three times a month. The AI raised migraine as a diagnosis because the headache was moderate and slightly interfering with her life, but the doctor diagnosed it as TTHs because there was no vomiting and no significant interference with her life. **The patient’s medical questionnaire indicated that she had nausea and vomiting, but in fact the headache was not related to nausea and vomiting, and other illnesses such as chronic gastritis were suspected.**

The latter case was a 32-year-old male with intermittent headaches for the past 10 years. Their frequency was several times a year, but the presence or absence of clustering could not be determined from the questionnaire alone. The headache attack lasted about a day. The headache was painful around the left side of the eye, the back of the head, and the neck. There was gouging and cracking pain in the eyes, and the headache was pulsatile. There was nausea and sound sensitivity, as well as redness of the eyes, running tears, and nasal discharge. There was stiffness in the shoulders and flashes of light as an aura. The AI diagnosed TACs based on factors such as nasal discharge and redness of the eyes on the medical questionnaire. However, the specialist diagnosed migraines in this person based on the presence of a visual aura, exacerbation with body movement, and the fact that triptans were effective for their headaches.

## 4. Discussion

We externally validated the previously developed AI model’s performance using prospectively collected diagnoses of 59 patients with headache specialists. The accuracy and kappa for the ground truth were 94.92% and 0.65 (95%CI 0.21–1.00), respectively, suggesting that this AI has the same diagnostic capabilities as headache specialists. To our knowledge, this is the first report on the external validation of an AI headache diagnosis model.

The number of headache specialists in Japan is low, at 982 doctors for a population of 130 million in 2023, so non-headache specialists often treat headache patients. It is also true that even specialists sometimes have difficulty diagnosing headaches; the diagnosis rate of repetitive migraines that interfere with daily life is 86.7% even among headache specialists, but 24.6% for intractable chronic migraines [70]. Furthermore, among non-specialists, the accuracy of headache diagnosis is as low as 46% [65]. Therefore, there is a need to develop a system to efficiently make correct headache diagnoses. In the realm of headache medicine, AI has the potential to improve diagnostic accuracy significantly. Our previous research indicates that an AI algorithm can boost diagnostic precision even among non-specialists in headache disorders [65]. With the widespread occurrence of migraines and other types of headaches and the scarcity of specialized headache doctors, such technological aids could enable patients to obtain accurate diagnoses, alleviating the impact of severe headaches and potentially lessening the need for referrals to neurologists.

In Japan, the percentage of headache patients visiting a medical institution is as low as 30% [19], and many patients purchase OTC painkillers depending on their own headache self-diagnosis and the possibility of developing MOHs. If migraine patients who especially need specialized treatment can be identified among these headache patients, they can visit a hospital and receive appropriate treatment. For example, a patient could be diagnosed by AI at home using a smartphone and realize that he or she has migraine headaches, which could lead to medical attention. It is also possible that a non-specialist physician, with the help of AI, could properly diagnose migraines and refer the patient to a specialist. Therefore, if AI diagnosis can be proven to be as accurate as that of headache specialists, it will improve the accuracy of migraine diagnosis by non-headache specialists and also increase the rate of outpatient visits by headache patients.

Our study shows that the diagnostic accuracy of the AI’s diagnosis is very high, with a kappa of 0.9. If a headache specialist examines a patient using this AI diagnosis, he/she does not need to check the entire headache diary but can accurately diagnose the remaining 10% that the AI diagnosis determines to be challenging to determine, thereby accurately ascertaining the number of migraine headaches and accurately determining the effectiveness of treatment. This is expected to reduce the burden on physicians, shorten patients’ treatment times, and improve the quality of medical care. High sensitivity and specificity are useful for screening and educating many migraineurs, but they may miss certain headaches. It is possible to adjust the output of the AI to achieve the desired level of sensitivity and specificity. Other screening tools, such as SNOOP10 [71], could also be used to screen for dangerous secondary headaches. How to use this AI in combination with other screeners needs to be explored in the future.

Our model’s sensitivity was as high as 98.21%, but its specificity was not as high at 66.67%. High sensitivity is suitable for screening migraines. The specialist’s diagnosis may not always be correct, and as noted in the ICHD-3, all applicable headaches should be considered and diagnosed. In particular, migraines coexisting with TTHs may be overlooked, so our model may be useful in terms of picking up as many migraine patients as possible. 

Many AI-based headache diagnostic models have been reported (Table 3). However, not one of these has been externally validated. Therefore, overfitting cannot be ruled out. Compared to these, our study is significant because we are the first to examine the usefulness of our own AI model in prospective cases collected at other institutions.

In this report, we discuss AI models for diagnosis. However, other AI-related research in the headache area is also gaining momentum. AI models for predicting treatment efficacy are also being developed. As an illustration, extensive data gathered from a smartphone app designed for electronic headache diaries were used to assess the effectiveness of acute migraine medications as reported by patients. AI promises to enhance outcome predictions, such as forecasting migraine episodes, anticipating responses to treatments, and identifying which migraine patients may develop additional health conditions [72]. AI has also been used to predict the therapeutic effect of CGRP-related drugs [73]. An AI model for predicting outcomes of headache surgery was also developed [74]. An AI model that extracts the number of monthly headache days from electronic medical records using a large language model has also been reported [75]. In this way, AI is increasingly being developed around the world, not only for diagnosis but also for predicting treatment effects and supporting medical treatment. AI is expected to become a good partner in headache treatment in the future.

Screening tools, not AI, have been developed so far. These are based on statistical methods, but it is unknown which is better than decision tree-based AI. As famous screeners, the Three-Question Headache Screen [76], ID Migraine [77], and the Four-Item Migraine Screener [77] have been reported, and their sensitivity and specificity range from 0.75 to 0.90. It remains to be seen whether AI or these tools are better, as their performance is highly dependent on the target population and other factors. We plan to examine the usefulness of these screening tools in our next research project when we have more cases.

Reports on domain shifting are scarce. This is the flip side of the fact that AI is being actively developed, but its usefulness has not been tested against other populations. This suggests that most medical AI models, although they can be developed on a trial basis, are unlikely to make it onto the market as a product. Only 17 medical AI models have been approved in Japan as of 2021 (https://www.pmda.go.jp/files/000244149.pdf (accessed on 20 April 2024)). Many processes are needed to put medical AI on the market as a product. In the future, it may be necessary to develop a system that allows AI to be created on a trial basis to demonstrate how useful it can be and to implement it in society as soon as possible.

This study has some limitations. First is the lack of further generalizability. The results were derived from data obtained from some hospitals or clinics with headache specialists. It is still unclear what the diagnostic performance is like outside of Japan or in an environment where non-specialists examine patients. In other words, the possibility that a domain shift is still inherent cannot be ruled out. Second, due to the exclusive reliance on questionnaire sheets in developing this model, information regarding neurological symptoms, vital signs, or other medical histories was not obtained. High specificity for secondary headaches is crucial in a diagnostic tool to prevent the misdiagnosis of potentially life-threatening conditions [71,78]. Hence, there is a need to develop an AI-based diagnostic tool capable of gathering data on patients’ histories and neurological findings, as well as radiological and laboratory test results, to effectively rule out secondary headaches. Third, the validation cohort included many migraine patients and did not include patients with TACs or other conditions. Therefore, kappa’s 95% CIs are wide-ranging. This suggests that although a prospective patient population was obtained, an even larger validation with no bias in the dataset is needed. **We will prospectively collect more cases to include more types of headaches and perform the analysis again in the future.** Although the model created in our previous study was used [65], it is necessary to further tune the model based on the data obtained prospectively in this way and further validate how the sensitivity and specificity of the model should be set for clinical use. Finally, AI should be used as a screening tool and for diagnostic assistance. An experienced headache specialist is also indispensable, given the diagnosis of secondary headaches and comorbid headaches of multiple types.

## 5. Conclusions

We externally validated the AI model’s performance using prospectively collected data from 59 patients. The diagnostic performance was strong for migraines and MOHs; the sensitivity, specificity, precision, and F-values for migraines were 98.21%, 66.67%, 98.21%, and 98.21%, respectively. Overall, the accuracy and kappa for the ground truth were 94.92% and 0.65 (95%CI 0.21–1.00), respectively, suggesting that this AI model has the same diagnostic capabilities as headache specialists. This AI headache diagnosis model may resolve the issue of undiagnosed and undertreated headaches and help non-headache specialists.

## Figures and Tables

**Table 1:** Patient characteristics (ground truth).

	Training Data *	Test Data *	Validation Data
	n = 2800	n = 1200	n = 59
**Mean age (standard deviation)**	41.27 (17.69)	41.38 (18.27)	42.55 (12.74)
**Biological sex (%Female)**	64.80%	63.20%	86.67%
**Class 1: Migraines or MOHs**	1597 (57.03%)	708 (59.00%)	56 (94.92%)
**Class 2: TTHs**	522 (18.64%)	197 (16.42%)	3 (5.08%)
**Class 3: TACs**	244 (8.71%)	101 (8.42%)	0
**Class 4: Other primary headaches**	55 (1.96%)	30 (2.50%)	0
**Class 5: Other headaches (secondary)**	382 (13.64%)	164 (13.67%)	0

Abbreviations: TACs, trigeminal autonomic cephalalgia; TTH, tension-type headache; MOH, medication-overuse headache; *: used for AI model, as previously reported in our other article [65].

**Table 2:** Confusion matrix.

		Prediction by AI						Performance Index				
		Class 1: Migraine or MOH	Class 2: TTH	Class 3; TACs	Class 4: Other Primary Headaches	Class 5: Other Headache (Secondary)	Total	Accuracy	Sensitivity (Recall)	Precision	Specificity	*F*-Value
Ground truth by headache specialist	**Class 1: Migraines or MOHs**	55	0	1	0	0	56	98.21%	98.21%	98.21%	66.67%	98.21%
	**Class 2: TTHs**	1	2	0	0	0	3	66.67%	66.67%	100.00%	100.00%	80.00%
	**Class 3: TACs**	0	0	0	0	0	0	-	-	-	100%	-
	**Class 4: Other primary headaches**	0	0	0	0	0	0	-	-	-	100%	-
	**Class 5: Other headaches (secondary)**	0	0	0	0	0	0	-	-	-	100%	-
	Total	56	2	1	0	0	59	94.92%	-	-	-	-

Free-marginal kappa = 0.65 (95% confidence interval: 0.21–1.00). Abbreviations: TACs, trigeminal autonomic cephalalgias; TTH, tension-type headache; MOH, medication-overuse headache.

**Table 3:** Previous reports on AI-based headache diagnosis models.

Author	Year	Data Source	Output by the Model	Methods	Variables	Training Sample Number	Test Sample Number	Validation Sample Number	%Migraine	Accuracy	Sensitivity (Recall)	Specificity	Precision	*F*-Value
**Yin** [62]	**2015**	**The International Headache Center in Chinese PLA General Hospital**	Class 2: migraine or TTHs	**Case-based reasoning + genetic algorithm**	**81**	**676**	**222**	**Not performed**	**76.1%**	**93.0%**	**97.0%**	**79.2%**	**93.1%**	**95.0%**
**Walters** [61]	**2016**	**University students**	**Class 2: migraines or other headache disorders**	**Logistic regression**	**4**	**887**	**942**	**Not performed**	**9.4%**	**92%**	**94%**	**92%**	**64%**	**93%**
**Vandewiele** [63]	**2018**	Patients with headache at the Department of Neurology, Ghent University Hospital. **Migbase dataset**.	**Class 3: migraines, TTHs, TACs**	**Decision tree**	**Not described**	**849**	**-**	**32**	**Not described**	**98%**	**98%**	**98%**	Not described	Not described
**Kwon** [64]	**2020**	**Samsung Medical Center headache clinic**	**Class 5: migraines, TTHs, TACs, thunderclap headaches, epicranial headaches**	**eXtreme gradient boosting**	**75**	**1286**	**876**	**Not performed**	**68.5%**	**58.6% †**	**58.7% †**	**85.6% †**	**65.3% †**	**58.6% †,‡**
**Cowan** [60]	**2022**	**Patients at three academic headache centers**	**Class 2: migraines or other headache disorders**	**Decision tree**	**135**	**-**	**-**	**212**	**62%**	**92%**	**89%**	**97%**	**98%**	**93%**
**Katsuki** [65]	**2023**	**Headache Center, Tominaga Hospital**	**Class 5: migraines or MOHs, TTHs, TACs, other primary headaches, or secondary headaches**	**Light gradient-boosting machine**	**17**	**2800**	**1200**	**50**	**60.0%**	**90.0%**	**68.6%**	**95.0%**	**96.4%**	**88.1%**
**Katsuki** [66]	**2023**	**Sendai Headache and Neurology Clinic**	**Class 5: migraines or MOHs, TTHs, TACs, other primary headaches, or other headaches**	**Gradient-boosting classifier**	**22**	**4240**	**1818**	**Not performed.**	**79.7%**	**93.7% †**	**40.6% †**	**48.5% †**	**88.7% †**	**43.5% †,‡**
**Ours** [65]	**2023**	**Headache Center, Tominaga Hospital**	**Class 5: migraines or MOHs, TTHs, TACs, other primary headaches, or secondary headaches**	**Light gradient-boosting machine**	**17**	**2800**	**1200**	**59**	**89.83%**	**92.11% §**	**92.11% §**	**92.86% §**	**33.33% §**	**95.41% §**

Abbreviations: AI, artificial intelligence; TACs, trigeminal autonomic cephalalgias; TTH, tension-type headache; MOH, medication-overuse headache; † Calculated as a macro-average; ‡ *F*-value by definition A; § only for migraine diagnosis.

## Data Availability

The datasets used and/or analyzed during the current study are available from the corresponding author upon reasonable request.
